# Maternal Perceptions and Responsiveness to Cry in Armed Conflict Zones: Links to Child Behavior Problems

**DOI:** 10.1007/s10802-024-01207-8

**Published:** 2024-06-04

**Authors:** Kinneret Levavi, Tal Yatziv, Porat Yakov, Alison Pike, Kirby Deater-Deckard, Amnon Hadar, Guy Bar, Miron Froimovici, Naama Atzaba-Poria

**Affiliations:** 1 Department of Psychology, Ben-Gurion University of the Negev, Beer-Sheva, Israel; 2 Duet Center, Ben-Gurion University of the Negev, Beer-Sheva, Israel; 3 School of Psychology, University of Sussex, Brighton, UK; 4 Department of Psychological and Brain Sciences, University of Massachusetts Amherst, Amherst, MA USA; 5 Clalit Health Services, Beer-Sheva, Israel; 6 Fertility and IVF Unit, Faculty of Health Sciences, Soroka University Medical Center, Ben-Gurion University of the Negev, Beer-Sheva, Israel

**Keywords:** Armed conflict zone, cry Perception, Responsiveness to cry, Behavior Problems, Parenting

## Abstract

Crying is a typical infant behavior that activates parental caregiving behaviors, acting as “human alarms” important for the infant’s survival. When living under war-related threat, the auditory system may be sensitized given its importance for survival, potentially impacting maternal cry processing. Children living in armed-conflict zones are at increased risk for behavior problems, which may relate to both direct exposure and indirect effects through their parents’ perceptions and behaviors. This hypothesis was examined in a sample of mothers and their first-born children (aged 10–45 months) living in the Gaza vicinity area in Israel, chronically exposed to missile alarms (high-exposure; *n* = 45), and a comparison group (low-exposure; *n* = 86). Group differences in child behavior problems and maternal perceptions of and responsiveness to cry were investigated. A moderated indirect-effect of maternal cry perceptions on child behavior problems via maternal responsiveness to cry was examined. In the high-exposure group, children had more externalizing problems and mothers rated cries as more aversive. Maternal cry perception was indirectly related to child behavior problems via responsiveness to cry only in the high-exposure group: higher perceptions of cry as aversive or the child as distressed were related to faster responding to crying, and faster cry responsiveness was linked with fewer behavior problems. Results suggest that in armed-conflict zones with auditory warning signals, the parental caring system may be easily activated by cries due to the strong association between alarms and threat. Furthermore, children may need their mothers to react faster when feeling distressed, possibly because of the surrounding threat.

Globally, wars and political violence are still an integral part of the lives of millions of children (Belsky, 2008; Denov & Akesson, 2016; Feldman et al., 2013; Kadir et al., 2019). For these children, ongoing wars, military operations, and terrorism have been a reality in their lives since they were born. The current study focuses on mothers and young children in the unique condition of living in the context of the armed conflict in the Gaza vicinity, an area in the southern part of Israel, several kilometers from the border between Israel and the Gaza Strip. The population living in this area has been experiencing ongoing missile attacks within civilian surroundings for decades (prior to the 2023 War). For example, between 2018 and 2019, over 2,000 rockets have been launched from Gaza to this area (Israeli Home Front Command, 2020). To warn and protect the residents, alarms are sound to notify about approaching missiles, giving residents in this area between 7 and 15 s to find shelter, a particularly challenging task for parents of young children who are not independently mobile. Moreover, the proximity to the border also exposes residents to the sounds of Israeli military operations in Gaza (e.g., warplane movement and sounds of bombing). Consequently, residents of this area are likely to be more alert and attentive to sounds, and their auditory system may be more sensitive to noises that inform about risk (Metzner et al., 2018). In this study, we examine whether this exposure is related to parental processing of another type of auditory alarm in the context of parenting—***child cries***—and thereby to child adjustment.

Studies conducted around the world indicate that exposure to any type of armed conflict may bear severe consequences for children of all ages, ranging from experiencing stress and exhibiting behavior problems to developing psychopathological disorders, such as post-traumatic stress disorder (PTSD), depression, and anxiety (for a meta-analysis in the age range of 5–17 years, see Attanayake et al., 2009). For example, long-term negative effects were found among children (aged 5–8 years old) exposed to war in Croatia, including aggressive and prosocial behavior (Keresteš, 2006). Studies conducted in the Gaza vicinity indicate that between 33% and 38% of infants and preschool children (age range: 1.5-5 years) living in this area exhibit partial or full PTSD symptoms (Feldman et al., 2013; Feldman & Vengrober, 2011; Pat-Horenczyk et al., 2012); these are likely underestimates of the impact of exposure on young children, as trauma due to exposure to armed conflict may not necessarily present as distinct forms of psychopathology and can manifest in widespread difficulties in different developmental domains (Cohen & Shulman, 2019; Cummings et al., 2017), including poor sleep quality, eating difficulties, psychosomatic symptoms, and behavior problems (Dybdahl, 2001; Masten & Narayan, 2012; Slone & Mann, 2016; Yahav, 2011). This suggests that the effects of exposure to armed conflict should be assessed by addressing broad developmental problems (Alkhatib et al., 2007; Dybdahl, 2001; Masten & Narayan, 2012; Pat-Horenczyk & Schiff, 2019; Sadeh et al., 2008; Slone & Mann, 2016), including externalizing (e.g., aggressive behaviors) and internalizing problems (e.g., fear, anxiety, affect dysregulation, and social withdrawal; Alkhatib et al., 2007; Punamäki, 2002; Yahav, 2011; Zamir et al., 2020).

Exposure to violence and combat can have prolonged effects on young children’s maladjustment (Keresteš, 2006; Laor et al., 2001), which may be exaggerated under chronic exposure (Slone & Mann, 2016). In areas where the exposure is continuous and may last for years, such as in the Gaza vicinity, chronic exposure to armed conflict was found to have more negative implications on young children’s (0–6 years old) emotional development and rates of internalizing and externalizing problems, as compared to acute exposure (Lahad & Leykin, 2010; Pat-Horenczyk & Schiff, 2019; Pat-Horenczyk et al., 2013; Slone & Mann, 2016). A possible explanation is that under prolonged exposure, children and parents live for longer periods in an unpredictable and undefined environment, which may elicit chronic stress (Cohen & Shulman, 2019). As children’s first years are particularly formative for subsequent development, such early exposure to armed conflict may have a long-lasting effect on future developmental processes, milestones, and achievements (Chu & Lieberman, 2010; Lieberman, 2011). For example, Alkhatib and colleagues (2007) suggested that exposure to chronic armed conflict stress in toddlerhood may interfere with the normative developmental process of gaining autonomy and self-confidence (separation-individuation) characteristic of this age by elevating fears of separation from their caregivers.

Alongside the widespread impact of trauma on risk for child maladjustment, some children show resilience and do not exhibit mental health symptoms or other developmental difficulties (Cummings et al., 2017; Masten & Narayan, 2012). To understand patterns of risk and resilience, researchers have suggested applying a *process-oriented* perspective (Cummings et al., 2014, 2017): rather than focusing solely on child developmental *outcomes*, there is a need for a wider systemic approach that takes into account *processes* and *pathways* occurring in the various circuits of a child’s life that may buffer against or elevate the risk for child maladjustment (Feldman & Vengrober, 2011; Masten, 2017). As understanding how developmental processes are modulated by exposure to armed conflict at a very young age is crucial for supporting children’s well-being (Cohen & Shulman, 2019; Feldman & Vengrober, 2011), we focus on direct and indirect effects on child adjustment.

## Parenting as a Main Mediator in the Context of Exposure to Armed Conflict

Cumulative evidence shows that young children are affected not only by direct exposure to armed conflict, but also by the effect of exposure on their primary caregivers (Betancourt & Khan, 2008; Cummings et al., 2017; Masten & Narayan, 2012; Pat-Horenczyk et al., 2012; Qouta et al., 2008). In their systematic review, Slone and Mann (2016) found that among various studies conducted in war or armed-conflict zones with children aged 0–6 years, there was a strong link between child adjustment and parental functioning and mental health. Interestingly, child maladjustment was more strongly related to parental factors than the severity of exposure itself (Betancourt & Khan, 2008; Zamir et al., 2020).

Parenting in armed-conflict zones is challenging. Parents exposed to armed conflict are vulnerable to psychopathology, such as PTSD, depression, and anxiety (Devakumar et al., 2014; Feldman et al., 2013; Pat-Horenczyk et al., 2012). In the Gaza vicinity, because missile attacks are sporadic, parents are in chronic uncertainty and have to adapt to unexpected events repeatedly, putting them in a constant state of vigilance and alertness (Cohen & Shulman, 2019). This chronic load may lead to loss of mental resources and result in feeling helplessness, which can impair the parent’s ability to (1) offer secure states of mind to the child and (2) regulate their own emotions (Cohen & Shulman, 2019; Sagi-Schwartz, 2012).

Chronic exposure to armed conflict can disrupt family daily dynamics, communication patterns, and parent-child relationship (Pat-Horenczyk et al., 2013). Mothers living in conflict zones may be at higher risk for less optimal parenting behavior, potentially affecting their child’s adjustment. For example, parental coercive behaviors were found to mediate the link between maternal mental health and child behavior problems when living in an armed-conflict zone (Zamir et al., 2020). Aiming to understand the way prolonged exposure to armed conflict may impact parental perceptions and practices, and how these may affect child adjustments, the current study investigates a unique parental capacity that may be triggered and challenged when living in an area where parents and children need to count on auditory signals to protect themselves: parental reactions to child cry.

### A Child’s Cry: Parental Perception and Responsiveness

We propose that the potential for increased auditory sensitivity due to prolonged armed conflict exposure may influence mothers’ perceptions of and reactions to children’s daily behaviors. One of the most relevant child behaviors is crying, which can be perceived as an “internal alarm” that activates the auditory system (Barr et al., 2001; Zeskind & Lester, 1978) as well as fight-flight-or-freeze behaviors (Joosen et al., 2013). As will be further elaborated below, parents of infants need to be attuned to infant cries, as important communicative signals that infants use to convey their needs (Bell & Ainsworth, 1972; Bowlby, 1982). As mentioned above, people living in Gaza vicinity are also likely to be highly attentive to auditory signals, and specifically their auditory system may be more alert and sensitive to noises that provide information on risk (Metzner et al., 2018): with time and classical conditioning (Clark, 2004), sirens (or other sounds that resemble alarms) become threatening and invasive stimuli (Pat-Horenczyk et al., 2012). This chronic exposure to auditory threat signals and the need to be alert and attend to them may have an impact on parental processing of infant cries, potentially mediating the link between exposure and child adjustment in early childhood. To further examine this idea, the link between maternal perception of child cry, perceived behavioral responding to cry, and child behavior problems were examined among mothers and children with high versus low exposure to armed conflict.

Crying is a universal primary behavior used by children for initial communication with caregivers, designed to elicit caregiving behavior by activating the caregiver’s attachment system to enhances proximity and meet the child’s needs (Bell & Ainsworth, 1972; Bowlby, 1982). According to Attachment Theory, when a crying child receives a sensitive response from their caregiver, healthy internal working models are developed (Bretherton, 1985; Gustafson et al., 2017; Lahti et al., 2019). At the same time, hearing a crying child can evoke negative feelings (such as stress, anxiety, and helplessness) that may impair the ability to respond sensitively and appropriately (Barr et al., 2001; Hiraoka et al., 2019; Joosen et al., 2013b).

Cry processing can be conceptualized as having two dimensions: **perceptions of crying** (cognitions) and **responsiveness to crying** (behaviors). *Perceptions of crying* consist of two different components: (1) auditory aversiveness—an acoustic component that relates to the auditory experience, and represents individual differences in auditory sensitivity when listening to the sound of crying and the difficulty of hearing a cry (Donovan et al., 2005; Gustafson et al., 2017; Joosen et al., 2013a; Zeskind & Lester, 1978), and (2) perception of child distress—a psychological component that refers to how the sound of crying is interpreted, representing a parent’s concerns about his or her child’s physical or mental state, which may cause distress (Barr et al., 2001; Sadeh et al., 2016; Zeifman, 2003). *Responsiveness to crying* refers to a behavioral component that addresses the ways parents intervene or respond to their crying child. Perceptions of cries and behavioral responsiveness to them are interrelated (Gustafson et al., 2017); for example, Zeifman (2003) found that parents who perceived the crying as urgent were likely to intervene earlier when hearing their child cry.

Previous studies have linked parental perceptions and responsiveness to child cry with various factors, including parental characteristics and psychopathology, child characteristics, and environmental factors (Donovan et al., 2005; Hiraoka et al., 2019; Lahti et al., 2019; Riem et al., 2011; Zeifman, 2003). For example, mothers who reported more difficulties in interpreting their child’s internal experiences reported and exhibited lower tolerance of infant crying when interacting with an inconsolable infant simulator doll (Rutherford et al., 2015). In the context of threatening and dangerous environments, Joosen et al. (2013) found an increased biological activation of defensive strategies through a fight-or-flight response while listening to cry, and that perceptions of cries as threatening were related to more hostile interpretations of child behaviors and harsh parenting among mothers of 12-months-old infants.

Importantly, parental responsiveness to child crying in the first year of life has been associated with various child outcomes, such as distress, sleeping problems, and sensorimotor development (Bretherton, 1985; Kahn et al., 2018; Lahti et al., 2019; Sadeh et al., 2016), although some patterns appear contradictory in their directionality. On the one hand, from an attachment point of view, a quick parental response to child needs (as expressed by cry) is an appropriate co-regulation response to a signal aimed to elicit caregiving behavior (Sameroff, 2010). On the other hand, an immediate maternal response to child crying may be less regulated (Martin et al., 2020) and potentially mitigate the child’s ability to develop self-regulation, resulting in more child regulation difficulties (Kahn et al., 2018; Sadeh et al., 2016). This seemingly contradictory pattern may be related to the nature and urgency of the distress context, as well as the child’s developmental stage and ability to cope by themselves. In the context of exposure to external chronic stress, as children need closer care from their parents with more direct regulation (Brom et al., 2008), we suggest that a quick response would be more helpful, resulting in lower levels of child problem behavior.

## The Current Study

Mothers who live in the Gaza vicinity have been suffering from chronic stress due to the armed conflict in this area, and are exposed to auditory stimulation (e.g., sirens) signaling upcoming danger (missile attack), such that many sounds become threatening and invasive (Pat-Horenczyk et al., 2012). We hypothesize that for mothers chronically exposed to warning alarms (“auditory stress”), compared to mothers with low exposure to auditory stress, a child’s cry may be associated with danger and thus may bias maternal perception and responsiveness to the cry and subsequently affect child behavior. To test this hypothesis, differences in maternal cry perceptions and responsiveness to cry, as well as in child behavior problems, were investigated in an Israeli sample with two groups experiencing high- versus low-exposure to armed conflict. Moreover, to better understand the underlying processes of child behavior problems in the context of armed conflict, an indirect effects model proposing that maternal perception would be associated with child behavior problems through maternal responsiveness to cry was examined for each group in a moderated-mediation model. We proposed that for the high-exposure group, the indirect effects model would be more robust when compared to the low-exposure group because children would be more vulnerable and may need their mother’s response to be immediate in order to better regulate.

### Study Hypotheses

**Fig. 1:**
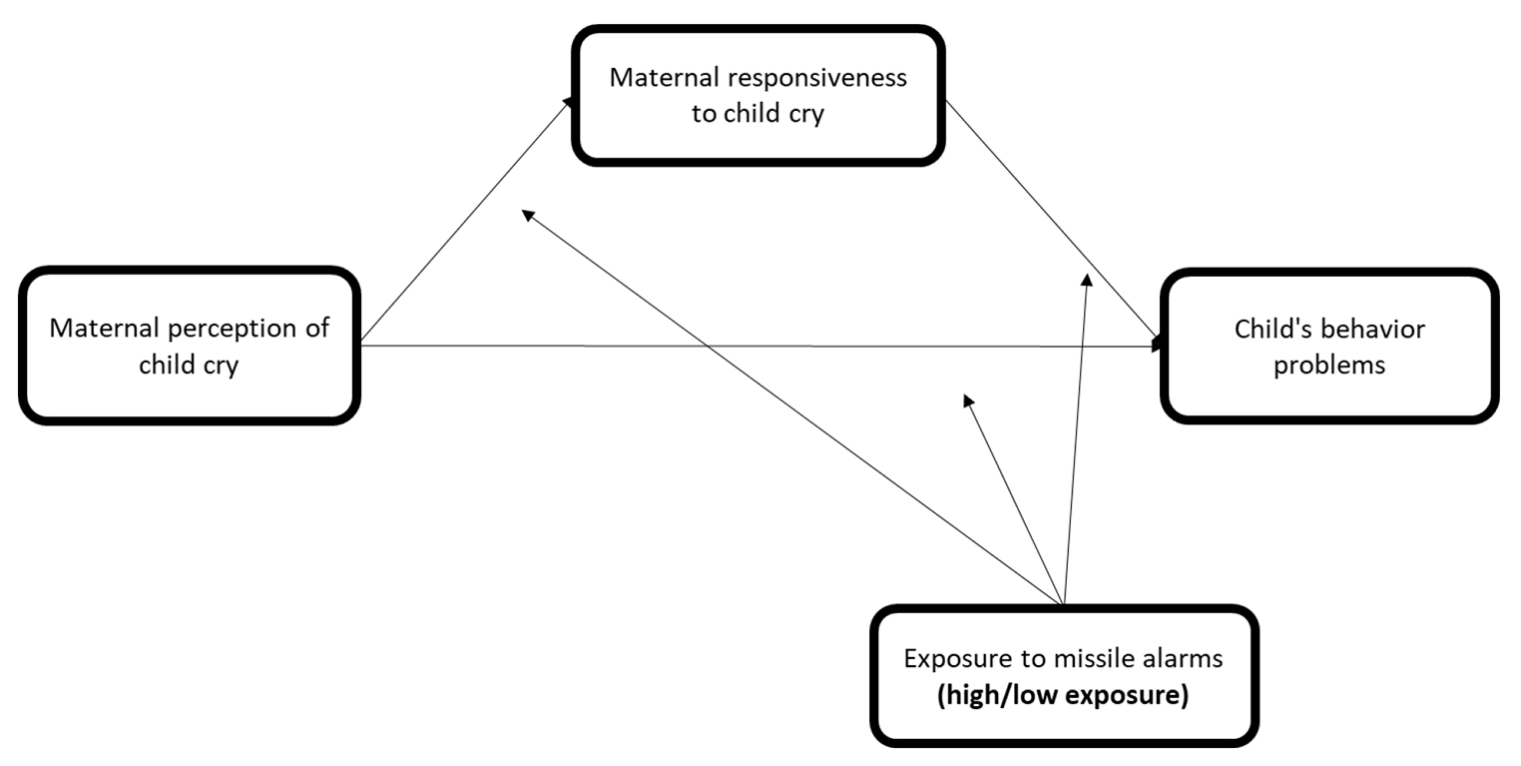
Note: Conceptual model of hypothesized direct and indirect effects

1. Group differences in **child behavior problems**: children in the high-exposure group would exhibit higher levels of externalizing and internalizing problem behaviors, compared to children in the low-exposure group.
2. Group differences in **maternal perception** and **maternal responsiveness** to child cry: mothers in the high-exposure group would report more difficulty listening to cries (auditory perception), attribute more distress to the child (psychological perception), and respond faster to the child, compared to mothers in the low-exposure group.
3. Moderated-mediation: The link between maternal perception of child cry and child externalizing and internalizing problem behaviors would be explained by maternal responsiveness to cry. That is, mothers who perceive the cry as more aversive, would tend to respond faster to child cry, and in turn, their children will show lower levels of behavior problems. Furthermore, we hypothesize that these associations would be stronger in the high-exposure group versus the low-exposure group, as cries may be especially important auditory signals to respond to in the context of war-related threat (see Fig. 1).

## Method

### Participants

Participants included 131 mothers and their firstborn children (10–45 months of age; 51.9% boys) from the southern district of Israel, who have been taking part in the longitudinal “Three to Four Study” examining familial changes upon the arrival of the second born. The current study includes data from the first time point, when mothers were pregnant, and had one child at home. The exposure groups were defined by their distance from the Gaza Strip, with the high exposure group (*n* = 45) including families living in localities within a range of up to 10 km from the Gaza Strip (Gaza vicinity). The low exposure group (*n* = 86) included families living in other areas in the south district of Israel. During the data collection period (October 2018 – March 2020, prior to the 2023 War), thousands of rockets were launched at towns in the Gaza vicinity, and they have become more frequent and unpredictable. These “rounds” of escalations and missile attacks were sometimes characterized by hundreds of rockets launched a day from the Gaza Strip and constant auditory alerts. Inclusion criteria included co-habiting two-parent heterosexual families (mother and father living together) expecting their second child, singleton pregnancies, typically-developing firstborns, and parents who were fluent in Hebrew. The high-exposure group was smaller mostly due to the challenges of conducting home visits in the area because of repeated missile attacks during the study period.

Demographic information concerning children, mothers, as well as the home crowding index (as an indicator for socioeconomic status [SES]) are detailed for each group in Table 1. Most of the mothers in the sample had high education levels and were, on average, 29 years old. Most mothers were born in Israel (85.5%), and the remaining were born in, or immigrated from, countries formerly in the Soviet Union (*n* = 11), or the UK, US, Germany, or Argentina (*n* = 1 each). It should be noted that unlike other samples often explored in conflict zones, commonly characterized by having low SES, being refugees, or being under other extreme situations in addition to the armed conflict exposure, the current study investigates an otherwise low sociodemographic-risk population in terms of having a typical life-style characteristic of the general Israeli society. However, occasionally this population is exposed to missile threats that violate the usual peace of their lives. The groups only differed in child age, such that children in the high-exposure group were younger than children in the low-exposure group, *t*(129) = -2.80, *p =*.006. Thus, age was controlled for in all analyses. No associations were found between demographic variables and other study variables.

**Table 1:** Descriptive Statistics for Demographic Variables by Exposure Groups (High/Low Exposure)

		Total sample (*n* = 131)	High-exposure group (*n* = 45)	Low-exposure group (*n* = 86)
Child gender (%)	Male	51.9%	46.7%	54.7%
	Female	48.1%	53.3%	45.3%
Child age (months)	Mean (SD)	24.66 (7.41)	22.22 (6.97)	25.94 (7.36)
Maternal age (years)	Mean (SD)	29.52 (3.99)	29.26 (3.8)	29.66 (4.11)
Maternal education (%)	Under 12 years	9.2%	6.7%	10.5%
	Nonacademic secondary education	10.7%	22.2%	4.7%
	Academic education	80.20%	71.1%	84.9%
Home-crowding index		0.94 (0.34)	1.02 (0.34)	0.90 (0.34)

### Procedure

The study was approved by the Clalit Helsinki Review Board and Ben-Gurion University of the Negev’s Human Subjects Research Committee. Mothers were recruited through women’s health centers, kindergartens, and online advertisements. Home visits were conducted with interested families. Informed consent was obtained from all mothers at the beginning of the home visit. During the home visit, mothers performed two computerized tasks concerning child crying and completed a questionnaire booklet. Other maternal and child measures out of the scope of the current analyses were taken as well.

### Research Tools

#### Maternal Perception of Child Cry

To measure maternal perception of child cry, the Ratings of Infant Crying Audio Segments (RICAS; Sadeh et al., 2016) was used. To our knowledge, parental perceptions of child cry using actual sounds of cry have not been tested in the context of exposure to armed conflict to date, and we therefore used an adapted scale that has been previously used in infant sleep studies (Sadeh et al., 2016). In the RICAS computerized procedure, mothers were presented with five 30-second audio recordings of a child’s cry with varying levels of intensity and tone. Following each recording, mothers were asked to rate the extent to which they agreed with five statements regarding their perception of the cry, on a 10-point Likert-like scale (“1- do not agree at all” to “10- very much agree”): (1) “*it was difficult to tolerate the sound of this cry*;” (2) “it *was difficult for me to hear a baby crying like this*;” (3) “*this crying baby is in distress*;” (4) *“this cry sounded spoiled or manipulative*” (reverse scored; low scores are indicative of a less hostile parental perception of the baby’s crying and are associated with a heightened perception of authentic distress in the infant); and (5) “*this cry sounded urgent and alarming*”. There were no missing data for this measure.

Consistent with previous research, a principal component analysis (PCA, varimax rotation) revealed that the five scales were loaded on two components (Sadeh et al., 2016). An examination of the Kaiser-Meyer Olkin measure of sampling adequacy suggested that the sample was factorable (KMO = 0.632): (1) the first component, “auditory aversiveness” included item 1 (“*It was difficult to tolerate the sound of this cry*”) and item 2 *(“It was difficult for me to hear a baby crying like this*”; loading ranged between: 0.83 and 0.90); (2) the second component, “perception of child distress” included item 3 (“*this crying baby is in distress*”), item 4 *(“This cry sounded spoiled or manipulative*”- reversed score), and item 5 *(“This cry sounded urgent and alarming*”; loading ranged between 0.71 and 0.79). These two principal components were used to test maternal perception to child cry.

#### Maternal Responsiveness to Child Cry

To assess maternal behavioral responsiveness to child cry, the Intervention Delay to Child Crying Video (IDICV; Sadeh et al., 2016) was used. In this procedure, mothers were told that they would be watching a short video of a baby crying. A cover story was given prior to watching the video: “The following video is of a very demanding baby. His parents are trying to ignore some of his crying to allow him to calm down by himself. Please look at the video and decide when you feel it’s absolutely necessary to intervene.” Then, mothers watched a 2-minute video clip of a 6-month-old infant playing on a carpet (for 10 s) who then starts crying, with a gradual increase in crying intensity and visual distress signs. The IDICV is coded as mothers’ intervention delay (in seconds). Five mothers were missing IDICV data.

### Child Behavior Problems

To measure child behavior problems, the Child Behavior Checklist for children aged 1.5-5 years (CBCL; Achenbach & Rescorla, 2000) was used. Mothers were asked to rate their children’s behaviors on a 0- to 2-point scale. Five scales were used for the current study assessing child’s internalizing behavior problems (24 questions, e.g., “unhappy, sad, or depressed”; Cronbach’s α = 0.86) and externalizing behavior problems (24 questions, e.g., “temper tantrums or hot temper”; Cronbach’s α = 0.89). Ten participants scored above the internalizing adjusted clinical cutoff and 10 scored within the borderline-clinical range, indicating relatively high prevalence of internalizing symptoms in this sample. In the externalizing subscale, one participant scored above the clinical cutoff and five scored within the borderline-clinical range, indicating that externalizing levels were not generally elevated in this sample. For 19% of the sample, CBCL data were missing due to technical issues.

### Analytic Plan

First, missing data was completed using an expectation-maximization filling method (EM algorithm; Newman, 2014) and preliminary analyses examining bivariate intercorrelations between study variables were conducted. Next, the first hypothesis, testing group differences in child behavior problems was conducted using an analysis of covariance (ANCOVA). Following, the second hypothesis examining group differences in maternal perception and responsiveness to cry was tested using three ANCOVA tests. To test the third hypothesis proposing that maternal perception of child cry will be associated with child behavior problems, directly and indirectly by maternal responsiveness and that this link would vary for the two exposure groups, a moderated-mediation model was tested using the PROCESS macro (Hayes, 2013; Model 59), with 95% bias-corrected bootstrapped confidence interval (CI) based on 2,000 bootstrap samples. Because maternal perception of child cry consisted of two distinct factors (auditory aversiveness and perception of child distress) and child behavior problems consisted of two different clusters (internalizing and externalizing behaviors), four separate models were tested. Continuous variables were first standardized. CIs that did not include zero indicated significant effects. Child age was controlled for in all the main models testing Hypotheses 1–3. Effect sizes of *r* =.10, 0.20, 0.30, and 0.40 were interpreted as reflecting small, medium, large, and very large effects, respectively (Funder & Ozer, 2019).

## Results

### Preliminary Analysis

Bivariate correlations between all study variables are presented in Table 2. As seen, there was a significant medium-to-large positive correlation between auditory aversiveness and perception of child distress for the entire sample, such that mothers who reported higher levels of auditory aversiveness also perceived the child as in more distress. Moreover, auditory aversiveness and perceptions of child distress were both negatively related to maternal responsiveness to cry (with medium and very large effect sizes, respectively), such that mothers who reported higher levels of auditory aversiveness and perceived the child as in more distress responded faster to the child’s cry. Child internalizing and externalizing behaviors were very strongly correlated.

**Table 2:** *Correlations Between Study Variables*

Variable	1.	2.	3.	4.
1. Auditory aversiveness	-			
2. Perception of child distress	0.28**	-		
3. Maternal responsiveness	− 0.20*	− 0.45**	-	
4. Internalizing behaviors	− 0.09	− 0.00	0.16	-
5. Externalizing behaviors	− 0.06	0.05	− 0.02	0.62**

Note. *p < .05, **p < .01

### Hypothesis 1: Group Differences in Child Behavior Problems

To test the first hypothesis proposing group differences between the exposure groups in child internalizing and externalizing problems, ANCOVA tests were conducted while controlling for child age. These analyses revealed significant differences in externalizing problems with a medium effect size, *F*(1,128) = 5.17, *p* =.025, *η*_*p*_^*2*^ = 0.04, such that in the high-exposure group, children exhibited more externalizing behavior problems than children from the low-exposure group (see Table 3). No significant difference was found for internalizing behavior problems, *F* < 1.

**Table 3:** *Means and Standard Deviation of Study Variables in the Two Exposure Groups*

Variable, M(SD)	High-exposure group (*n* = 45)	Low-exposure group (*n* = 86)
1. Auditory aversiveness	6.07 (1.52)	5.51 (1.56)
2. Perception of child distress	5.45 (1.04)	5.46 (0.95)
3. Maternal responsiveness	34.67 (10.54)	36.43 (11.27)
4. Internalizing behaviors	0.24 (0.16)	0.21 (0.18)
5. Externalizing behaviors	0.48 (0.18)	0.39 (0.23)

### Hypothesis 2: Group Differences in Maternal Perception of and Responsiveness to Child Cry

The second hypothesis proposed group differences in maternal perception to child cry (both auditory aversiveness and perception of child distress) and maternal responsiveness, such that in the high-exposure group mother would perceive child cries as more aversive and reflecting higher child distress, as well as quicker response to the child’s cry. To test these hypotheses, three ANCOVA tests were conducted, controlling for child age. Differences in auditory aversiveness approached significance with a small effect size, *F*(1,128) = 3.36, *p* =.069, *η*_*p*_^*2*^ = 0.03, with mothers in the high-exposure group reporting higher levels of auditory aversiveness than mothers in the low-exposure group (see Table 3). No significant differences between the groups were found for perception of child distress or maternal responsiveness, *Fs* < 1.

### Hypothesis 3: Direct and Indirect Effects by Groups

To test the third hypothesis proposing that maternal perception of child cry will be associated with child behavior problems directly, and indirectly via maternal responsiveness to cry, and that this link would vary for the two exposure groups, a moderated-mediation model was tested (Fig. 1). This analysis tested whether exposure group (high/low) moderated the proposed indirect effects model (i.e., maternal perception of child cry predicts maternal responsiveness to child cry, and in turn, child behavior problems). Results are presented in Tables 4 and 5 for either auditory aversiveness and perception of child distress as the focal predictor, respectively. Exposure group did not interact with maternal cry perception (averseness or distress) in relation to responsiveness to cry; however, exposure group moderated the link between maternal responsiveness to cry and child symptoms.

**Table 4:** *Results of the Moderated-Mediation Analysis Presenting Direct and Indirect Effects of Auditory Aversiveness as an Independent Variable*

Mediator variable model (IDICV)			b (SE)	t	*p*			
Auditory aversiveness			-1.36 (0.62)	-2.20	0.030			
Exposure group			0.46 (2.07)	0.22	0.826			
Auditory aversiveness x Exposure group			1.28 (1.32)	0.97	0.334			
Child age			0.11 (0.13)	0.84	0.402			
***Dependent Variable Model *** ***(Behavioral Problems)***			**Dependent Variable: Internalizing Problems**			**Outcome Dependent: Externalizing Problems**		
			***b (SE)***	***t***	***p***	**b** ***(SE)***	***t***	***p***
IDICV			0.002 (0.00)	2.02	0.045	0.0001 (0.002)	0.08	0.936
Auditory aversiveness			-0.01 (0.01)	-0.73	0.467	-0.01 (0.01)	-0.98	0.329
IDICV x Exposure group			-0.01 (0.00)	-2.07	0.041	-0.01 (0.004)	-1.98	0.049
Exposure group			-0.04 (0.03)	-1.15	0.25	-0.1 (0.04)	-2.4	0.017
Auditory aversiveness x Exposure group			-0.02 (0.02)	-0.86	0.391	-0.02 (0.03)	-0.80	0.425
Child age			-0.00 (0.00)	-0.92	0.359	-0.002 (0.003)	-0.70	0.485
***Conditional Indirect Effect at Specific Levels of Exposure Groups***								
Mediator	Moderator (group)		**Indirect effect** ***(SE)***	**LL 95% CI**	**UL 95% CI**	**Indirect effect** ***(SE)***	**LL 95% CI**	**UL 95% CI**
IDICV		High exposure	-0.01 (0.01)	-0.04	-0.0005	-0.01 (0.01)	-0.04	-0.001
		Low exposure	-0.0006 (0.002)	-0.01	0.002	0.002 (0.003)	-0.001	0.01

***Notes***. *N* = 131

**Table 5:** *Results of the Moderated-Mediation Analysis Presenting Direct and Indirect Effects of Perception of Infant Distress as an Independent Variable*

Mediator Variable Model (IDICV)			b (SE)	t	*p*			
Perception of child distress			-4.93 (0.89)	-5.54	< 0.01			
Exposure group			1.5 (1.86)	0.81	0.419			
Perception of infant distress x Exposure group			0.69 (1.83)	0.38	0.704			
Child age			0.11 (0.12)	0.92	0.359			
***Dependent Variable Model *** ***(Behavioral problems)***			**Outcome Variable: Internalizing Problems**			**Outcome Variable: Externalizing Problems**		
			***b (SE)***	***t***	***p***	***b (SE)***	***t***	***p***
IDICV			0.004 (0.001)	2.60	0.010	0.001 (0.002)	0.72	0.473
Perception of child distress			0.02 (0.02)	1.17	0.244	0.02 (0.02)	0.90	0.370
IDICV x Exposure group			-0.01 (0.003)	-2.62	0.010	-0.01 (0.004)	-2.47	0.015
Exposure group			-0.04 (0.03)	-1.24	0.217	-0.10 (0.04)	-2.45	0.016
Perception of infant distress x Exposure group			-0.06 (0.04)	-1.56	0.121	-0.06 (0.04)	-1.41	0.161
Child age			-0.002 (0.002)	-1.11	0.269	-0.002 (0.003)	-0.82	0.414
***Conditional Indirect Effect at Specific Levels of Exposure Groups***								
Mediator		Moderator	**Indirect effect** ***(SE)***	**LL 95% CI**	**UL 95% CI**	**Indirect effect** ***(SE)***	**LL 95% CI**	**UL 95% CI**
IDICV	High exposure		-0.05 (0.02)	-0.12	-0.02	-0.04 (0.02)	-0.10	-0.01
	Low exposure		-0.004 (0.01)	-0.02	0.001	0.01 (0.01)	-0.01	0.03

*Notes*. *N* = 131

**Fig. 2:**
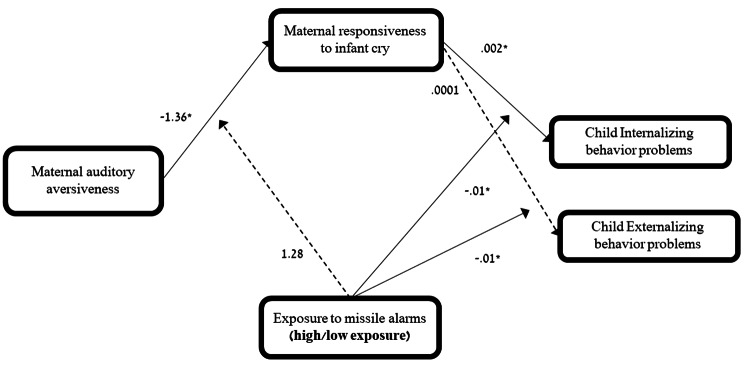
*Notes*. Regression coefficients for the moderated-mediation model testing whether exposure group moderated the direct and indirect effects of maternal auditory aversiveness in statistically predicting internalizing/externalizing behavior problems via maternal responsiveness to cries. Full lines represent significant paths, whereas dashed paths are nonsignificant. * *p* <.05

Supporting the third hypothesis, results indicated an indirect link between **auditory aversiveness** and child internalizing problems via maternal responsiveness to cries, only in the high-exposure group but not in the low-exposure group (see Table 4; Fig. 2). Similar results were found for child externalizing problems, indicating an indirect path from auditory aversiveness via maternal responsiveness to cry only in the high-exposure group, and not in the low-exposure group (see Table 4; Fig. 2). That is, in the high-exposure group, the more difficulty a mother reported listening to the child crying, the faster she responded, and the less internalizing and externalizing behavior problems her child showed.

**Fig. 3:**
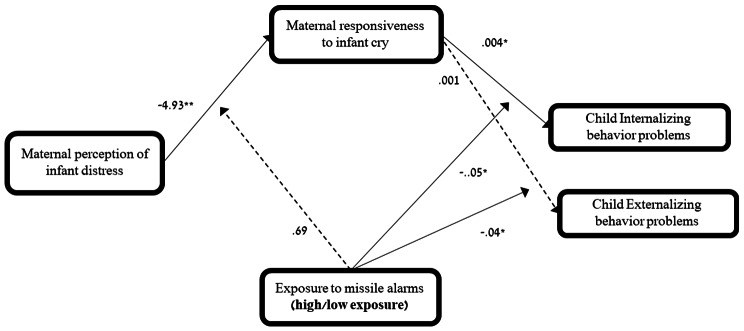
*Notes*. Regression coefficients for the moderated-mediation model testing whether exposure group moderated the direct and indirect effects of maternal perception of infant distress in statistically predicting internalizing/externalizing behavior problems via maternal responsiveness to cries. Full lines represent significant paths, whereas dashed paths are nonsignificant. * *p* <.05, ** *p* <.01

Results also revealed an indirect link between **perception of child distress** and child internalizing problems via maternal responsiveness to cries only in the high-exposure group, and not in the low-exposure group (see Table 5; Fig. 3). Similarly, an indirect path from perception of child distress to externalizing problems via maternal responsiveness to cry was evident only in the high-exposure group, and not in the low-exposure group (see Table 5; Fig. 3). Thus, results suggest that for the high-exposure group, the more distress a mother attributed to the child’s crying, the faster she responded, and in turn, the less internalizing and externalizing behavior problems were reported.

## Discussion

Children growing up in armed-conflict zones are at risk for maladjustment, not only through direct exposure to danger but also due to parental perceptions and behaviors that may be affected by the stressful, external, situation. In the Gaza vicinity, there is a unique situation in which families (who are otherwise representative of the general population) have been under unpredictable missile attacks for many years, placing them at high risk to their safety and security. Because these attacks are accompanied by alarms that warn the population, a high sensitivity to sounds indicating danger is crucial and can save lives. Therefore, the objectives of the current study were to examine whether children who are exposed to armed conflict over time are at higher risk for exhibiting behavior problems, and how parental perceptions and practices concerning cry are linked to child behavior problems.

The first hypothesis proposing group differences in child behavior problems was partially supported. Differences between the exposure groups in child behavior problems were found for externalizing behaviors but not for internalizing behaviors. Although significant, the effect size was small and should be interpreted with caution. Nonetheless, this pattern is consistent with past research (Slone & Mann, 2016), finding that children under high exposure to armed conflict have more externalizing behavior problems compared to children under low exposure, especially when exposed chronically like in the present sample.

In contrast, the groups did not differ in child internalizing problems, which is inconsistent with previous research commonly using the same scale (for a review, see Slone & Mann, 2016). Although this null effect should be interpreted with caution, the lack of effect may be related to the young age of the children and the unique characteristics of the current sample. First, children between the ages of 2–5 years tend to exhibit elevated internalizing behaviors on average (Gilliom & Shaw, 2004; Mathiesen et al., 2009), which may dilute group differences. Second, mothers in the high-exposure group may be biased in their reporting of behaviors indicative of internalizing problems, such as clinging, separation anxiety, sudden changes in mood, and constant anxiety, as these may be perceived as “normal” reactions or behaviors when living under threatening conditions, and therefore mothers might not regard them as symptoms in their reporting. Finally, previous theories suggest that internalizing problems may arise in children in the context of emotionally unsupportive parenting (Gilliom & Shaw, 2004). As the current sample was characteristic of the general population in all other relevant aspects (e.g., low-risk in SES and social characteristics), it is likely that children received a supportive and safe care overall, even under chronic exposure to stress, and thus their internalizing symptoms were not affected.

The second hypothesis proposing group differences in maternal cry perceptions and responsiveness was partially confirmed. A significant difference between the groups was found for auditory aversiveness, but not for perception of child distress or maternal responsiveness to cry. These findings strengthen our assumption that mothers in the high-exposure group were more sensitive to the auditory-sensory aspect of the crying sound, possibly because they were more sensitized to sounds that may indicate danger. These results may suggest that parents who live in armed-conflict areas, characterized by auditory warnings, may develop a hypersensitivity to sounds, even when they know that some of the sounds they hear are not related to danger. Furthermore, it is known that the sound of crying is a particular sound that triggers the parental caring system (Bell & Ainsworth, 1972) and thus ensures a child’s survival. Our results raise the idea that when living in an armed-conflict zone, the parental caring system may be more easily activated due to the strong association between the sounds of the alarm and an imminent threat. As a result, mothers may develop high sensitivity to the sound of crying. Although significant, the effect size was small and thus should be interpreted with caution.

It is interesting to note that no significant differences were found for perception of child distress or maternal responsiveness to cry between the groups. It is possible that the impact of exposure may be primarily related to feelings elicited in the mother by the cries but may not impact the aspects of cry processing centered around the child’s state. Alternatively, the high-exposure groups’ sociodemographic characteristics may have impacted this as well, as despite living in an armed-conflict area, the sample had low social risk, resembling (in some aspects) parents in low-exposure areas.

Moving beyond group differences, our second aim was to investigate underlying processes in the relation between maternal factors and child outcomes in the context of exposure to armed conflict. Specifically, our third hypothesis addressed the indirect link between maternal cry perception and child behavior problems via maternal responsiveness to crying and examined whether the links varied by exposure group. Indeed, this indirect effect models were found to be significant, such that maternal perception of cry was associated with child behavior problems (both externalizing and internalizing problem behaviors) through maternal responsiveness to cry, *only in the high-exposure group*. Mothers who perceived the crying sounds as more aversive or perceived the child as more distressed responded more quickly to the cry. In the high-exposure group, when mothers responded faster, their children showed fewer behavior problems compared to mothers who responded slower to the crying sound. The link between maternal responsiveness and child behavior problems was not evident in the low-exposure group.

These findings are consistent with previous work that emphasized the important role of maternal factors as key elements in understanding child behavior problems when living in an armed-conflict zone (Zamir et al., 2020). From an attachment point of view, the parent serves as a “safe haven” to which the child can return when experiencing distress (Bretherton, 1985). Because very young children are mostly dependent on their parents to regulate their emotions (Sameroff, 2010), maternal response to a child’s cry may be crucial for the child’s emotional regulation especially under external stressful situation (Brom et al., 2008). Hence, when growing up in an environment in which missile attacks are part of everyday life, and warnings of the attacks comes are auditory (i.e., an alarm) and require an immediate reaction (i.e., 7–15 s), a maternal quick response may be lifesaving and is required when children experience fear and danger. Thus, a pattern of coping and mutual self-regulation based on prompt response is created (Pat-Horenczyk et al., 2015; Sameroff, 2010). Furthermore, it seems that the need for an immediate maternal response, as seen by its association with child adjustment, is not limited only to the mother’s response during an external warning alarm, but may be generalized to more normative, everyday situations where her child expresses distress through crying.

It should be noted that in other studies, a less prompt response to infant cries was sometimes considered more adaptive (Kahn et al., 2018; Martin et al., 2020; Sadeh et al., 2016). A possible explanation for the differences in the results is that in the context of an armed-conflict zone, mothers who react more quickly may provide their children with a better sense of protection that may, in turn, help them to regulate their fears. However, when living in a safe environment, an immediate parental reaction to a child’s cry may interrupt the developmental process of self-regulation and mitigates a child’s opportunity to regulate themselves independently (Kahn et al., 2018; Sadeh et al., 2016). The developmental change from co-regulation to self-regulation (see Sameroff, 2010) may thus require a longer period of co-regulation when living under stress. Thus, if a mother in the high-exposure group does not respond immediately to her child’s needs, the child may not be able to independently cope with distress without support, as seen in elevated levels of behavior problems. Our results corroborate the ideas of Feldman and Vengrober (2011), who suggested that avoidance behaviors in children growing up in an armed-conflict context may indicate that the mother failed to contain the child’s anxieties, resulting in behavior difficulties.

Yet, the question arises whether these patterns of regulation and dependency between mothers and children are adaptive over time, or might increase the risk for developmental difficulties in later stages of development. This question is particularly salient when children are exposed to risk during the separation-individuation stage (Mahler & La Perriere, 1965), at the age of 1–3 years (as in the current sample). The main developmental task of children around this age is to develop independence. Studies on infants and toddlers exposed to trauma indicate that the external risk may interfere with the development of trust and autonomy (Slone & Mann, 2016). Thus, whether maternal behaviors of immediate reaction to a child’s cry, which seems to be related to better child adjustment during the age of 1–3 years, may have some negative consequences in the future, remains to be tested. More specifically, will children in the high-exposure group exhibit more dependency on their caregivers as they get older? Further longitudinal studies are required to track these developmental paths.

When discussing the complex situation in the Gaza vicinity, cultural features of the population should be taken into consideration. Most families living in this area identify as Jewish-Israeli, and some of the families reside there out of choice. Families living in the Kibbutzim often describe the quietness, beauty, and serenity of this region during calmer times. Such attitudes may interact with exposure to threat, and potentially be a buffer, influencing how they psychologically react to the chronic stress they experience due to the ongoing conflict in the region. Understanding and respecting these cultural perspectives are essential when considering interventions and support mechanisms for these families facing such challenging circumstances. In addition, due to the nature of the study’s population the findings of the present study may not be generalized to other populations suffering from armed conflict until replicated. That is, on the one hand, the current sample is characterized overall as a typical sample within the Israeli population. On the other hand, these families have been suffering from prolonged exposure to threat and experience chronic stress due to other aspects of their demographics.

### Limitations, Future Directions, and Clinical Implications

This study offers new insights into pathways through which chronic exposure to armed conflict may relate to early mother-child relationships and development. Nonetheless, several limitations should be acknowledged. First, the current study is a cross-sectional study and therefore it is difficult to infer causality. Although we had theoretically-sound reasons to infer directionality, it is possible that child behavior difficulties are those that led mothers to perceive child cry in a more detached manner, as seen by a slower reaction, or that other factors intervene and affected the results. Replicating these findings in a longitudinal design would enable a deeper understanding of these links. Second, the sample size may be considered relatively small, especially in the high-exposure group, which may have impacted our ability to detect smaller effects. Third, this study referred to a group of mothers who had experienced chronic stress for many years, not only as a result of the auditory stress of the alarms, but also due to living in an active armed conflict zone. Thus, in future research it is important to examine additional aspects of objective and perceived stress and exposure and their effect on mothers’ perception and responsiveness to child cries.

Finally, it is important to note that this study had some specific characteristics that may limit generalizability to other populations: high-exposure families were living in a specific area, all mothers had only one child, and all were pregnant with their second born. Although group differences are not likely to be explained by pregnancy (given all mothers in both groups were pregnant with their second born), generalizability to non-pregnant mothers may be limited given the hormonal fluctuations that may affect pregnant women’s mood, social behavior, and emotional responses (Li et al., 2020). Future studies should replicate these findings with additional populations living under other types of chronic stress, and among pregnant and non-pregnant mothers, as well as fathers, to test whether they can they be generalized for other caregivers under other risk conditions.

Despite these limitations, the findings of the current study have clinical implications for working with families experiencing chronic war-related threat and developing interventions. Alongside emotional treatments given to children and adults separately, interventions focusing on early parent-child relationships may also assist in supporting child adjustment and mental health. Specifically, working with mothers and expectant mothers assisting interpretations of child cries and behavioral responsivity to crying may facilitate co-regulation and thereby alleviate child behavioral problems in response to chronic stress while living in a threatening environment, and potentially assisting the child in developing their own capacity to regulate and deal with the situation as they grow older. In families expecting a new child, planning ahead to ensure the availability of a caregiver to support the older child under distress (e.g., extended family or friends) may be especially beneficial for coping.

## Conclusions

Even though many children experience war-related threat across the world, studies regarding young children’s adjustment in the context of armed conflict, especially concerning mediating and moderating factors underlying developmental processes, are still scarce. The main goal of the current study was to uncover the role of maternal perceptions of and responsiveness to child cry in young children’s adjustment, comparing children in low- and high-exposure environments. Taken together, our results suggest that exposure to external war-related threat can have a spillover effect, affecting a mother’s sensitivity and reactions to normative day-to-day behaviors toward her children. This study provides initial foundations for future research to explore developmental trajectories and precursors of psychopathology when living under chronic stress, and the possible costs in child development.

## References

[CR1] Achenbach, T. M., & Rescorla, L. A. (2000). Manual for the ASEBA preschool forms & profiles: An integrated system of multi-informant assessment; Child behavior checklist for ages 1 1/2–5; Language development survey; Caregiver-teacher report form. *University of Vermont*.

[CR2] Alkhatib, A., Regan, J., & Barrett, D. (2007). The silent victims: Effects of war and terrorism on child development. *Psychiatric Annals*, *37*(8), 586–589.

[CR3] Attanayake, V., McKay, R., Joffres, M., Singh, S., Burkle, F., & Mills, E. (2009). Prevalence of mental disorders among children exposed to war: A systematic review of 7,920 children. *Medicine Conflict and Survival*, *25*(1), 4–19. 10.1080/13623690802568913. 19413154 10.1080/13623690802568913

[CR4] Barr, R. G., James-Roberts, I. S., & Keefe, M. R. (2001). New Evidence on Unexplained Early Infant Crying: Its Origins, Nature and Management. In *Johnson & Johnson Pediatric Round Table Series*. 10.1097/00005176-200201000-00026.

[CR5] Bell, S. M., & Ainsworth, M. D. S. (1972). Infant crying and maternal responsiveness. *Child Development*, *43*(4), 1171–1190. 4643768

[CR6] Belsky, J. (2008). War, trauma and children’s development: Observations from a modern evolutionary perspective. *International Journal of Behavioral Development*, *32*(4), 260–271. 10.1177/0165025408090969.

[CR7] Betancourt, T. S., & Khan, K. T. (2008). The mental health of children affected by armed conflict: Protective processes and pathways to resilience. *International Review of Psychiatry*, *20*(3), 317–328. 10.1080/09540260802090363. 18569183 10.1080/09540260802090363 PMC2613765

[CR8] Bowlby, J. (1982). Attachment and loss: Retrospect and prospect. *American Journal of Orthopsychiatry*, *52*(4), 664–678. 10.1111/j.1939-0025.1982.tb01456.x. 7148988 10.1111/j.1939-0025.1982.tb01456.x

[CR9] Bretherton, I. (1985). Attachment theory: Retrospect and prospect. *Monographs of the Society for Research in Child Development*, *50*(1), 3–35. 10.2307/3333824.

[CR10] Brom, D., Pat-Horenczyk, R., & Ford, J. D. (Eds.). (2008). *Treating traumatized children: Risk, resilience and recovery*. Routledge.

[CR11] Chu, A. T., & Lieberman, A. F. (2010). Clinical implications of traumatic stress from birth to age five. *Annual Review of Clinical Psychology*, *6*(1), 469–494. 10.1146/annurev.clinpsy.121208.131204. 20192799 10.1146/annurev.clinpsy.121208.131204

[CR12] Clark, R. E. (2004). The classical origins of Pavlov’s conditioning. *Integrative Physiological and Behavioral Science*, *39*(4), 279–294. 10.1007/BF02734167. 16295771 10.1007/BF02734167

[CR13] Cohen, E., & Shulman, C. (2019). Mothers and toddlers exposed to political violence: Severity of exposure, emotional availability, parenting stress, and toddlers’ behavior problems. *Journal of Child and Adolescent Trauma*, *12*(1), 131–140. 10.1007/s40653-017-0197-1. 32318186 10.1007/s40653-017-0197-1 PMC7163821

[CR14] Cummings, E. M., Goeke-Morey, M. C., Merrilees, C. E., Taylor, L. K., & Shirlow, P. (2014). A social-ecological, process-oriented perspective on political violence and child development. *Child Development Perspectives*, *8*(2), 82–89. 10.1111/cdep.12067. 26877765 10.1111/cdep.12067 PMC4749157

[CR15] Cummings, E. M., Merrilees, C. E., Taylor, L. K., & Mondi, C. F. (2017). Developmental and social–ecological perspectives on children, political violence, and armed conflict. *Development and Psychopathology*, *29*(01), 1–10. 10.1017/S0954579416001061. 27869066 10.1017/S0954579416001061

[CR16] Denov, M., & Akesson, B. (2016). Children and political violence: At the intersection of rights and realities. *Children and Society*, *30*(5), 337–344. 10.1111/chso.12175.

[CR17] Devakumar, D., Birch, M., Osrin, D., Sondorp, E., & Wells, J. C. K. (2014). The intergenerational effects of war on the health of children. *BMC Medicine*, *12*(1), 1–15. 10.1186/1741-7015-12-57. 10.1186/1741-7015-12-57 PMC3997818 24694212

[CR18] Donovan, W., Leavitt, L., & Taylor, N. (2005). Maternal self-efficacy and experimentally manipulated infant difficulty effects on maternal sensory sensitivity: A signal detection analysis. *Developmental Psychology*, *41*(5), 784–798. 10.1037/0012-1649.41.5.784. 16173875 10.1037/0012-1649.41.5.784

[CR19] Dybdahl, R. (2001). Children and mothers in war: An outcome study of a psychosocial intervention program. *Child Development*, *72*(4), 1214–1230. 11480943 10.1111/1467-8624.00343

[CR20] Feldman, R., & Vengrober, A. (2011). Posttraumatic stress disorder in infants and young children exposed to war-related trauma. *Journal of the American Academy of Child and Adolescent Psychiatry*, *50*(7), 645–658. 10.1016/j.jaac.2011.03.001. 21703492 10.1016/j.jaac.2011.03.001

[CR21] Feldman, R., Vengrober, A., Eidelman-Rothman, M., & Zagoory-Sharon, O. (2013). Stress reactivity in war-exposed young children with and without posttraumatic stress disorder: Relations to maternal stress hormones, parenting, and child emotionality and regulation. *Development and Psychopathology*, *25*, 943–955. 10.1017/S0954579413000291. 24229541 10.1017/S0954579413000291

[CR22] Funder, D. C., & Ozer, D. J. (2019). Evaluating effect size in psychological research: Sense and nonsense. *Advances in Methods and Practices in Psychological Science*, *2*(2), 156–168. 10.1177/2515245919847202.

[CR23] Gilliom, M., & Shaw, D. S. (2004). Codevelopment of externalizing and internalizing problems in early childhood. *Development and Psychopathology*, *16*(2), 313–333. 10.1017/s0954579404044530. 15487598 10.1017/s0954579404044530

[CR24] Gustafson, G. E., Bisson, J. B., Macdonald, J. M., & Green, J. A. (2017). Infant behavior and development a ff ective reactivity to cry sounds predicts young women ’ s reactivity and behavior in a simulated caregiving task. *Infant Behavior and Development*, 1–10. 10.1016/j.infbeh.2017.08.004. 10.1016/j.infbeh.2017.08.004 28917387

[CR25] Hayes, A. (2013). Integrating mediation and moderation analysis: Fundamentals using PROCESS. In *Introduction to Mediation, Moderation and Conditional Process Analysis*.

[CR26] Hiraoka, D., Miyasaka, M., & Nomura, M. (2019). Spousal presence modulates salivary α-Amylase responses to infant cry in mothers with high attachment insecurity. *Parenting*, *19*(1–2), 5–21. 10.1080/15295192.2019.1555416.

[CR27] Israeli Home Front Command (2020). *Alert History, Retrieved from* https://info.oref.org.il/12481-he/Pakar.aspx.

[CR28] Joosen, K. J., Mesman, J., Bakermans-Kranenburg, M. J., Pieper, S., Zeskind, P. S., & Van Ijzendoorn, M. H. (2013a). Physiological reactivity to infant crying and observed maternal sensitivity. *Infancy*, *18*(3), 414–431. 10.1111/j.1532-7078.2012.00122.x.

[CR29] Joosen, K. J., Mesman, J., Bakermans-Kranenburg, M. J., & van IJzendoorn, M. H. (2013b). Maternal overreactive sympathetic nervous system responses to repeated infant crying predicts risk for impulsive harsh discipline of infants. *Child Maltreatment*, *18*(4), 252–263. 10.1177/1077559513494762. 23836807 10.1177/1077559513494762

[CR30] Kadir, A., Shenoda, S., & Goldhagen, J. (2019). Effects of armed conflict on child health and development: A systematic review. *Plos One*, *14*(1), 1–37. 10.1371/journal.pone.0210071. 10.1371/journal.pone.0210071 PMC6334973 30650095

[CR31] Kahn, M., Bauminger, Y., Volkovich, E., Meiri, G., Sadeh, A., & Tikotzky, L. (2018). Links between infant sleep and parental tolerance for infant crying: Longitudinal assessment from pregnancy through six months postpartum. *Sleep Medicine*, *50*, 72–78. 10.1016/j.sleep.2018.05.014. 30015254 10.1016/j.sleep.2018.05.014

[CR32] Keresteš, G. (2006). Children’s aggressive and prosocial behavior in relation to war exposure: Testing the role of perceived parenting and child’s gender. *International Journal of Behavioral Development*, *30*(3), 227–239. 10.1177/0165025406066756.

[CR33] Lahad, M., & Leykin, D. (2010). Ongoing exposure versus intense periodic exposure to military conflict and terror attacks in Israel. *Journal of Traumatic Stress*, *23*(6), 691–698. 10.1002/jts.20583. 21171129 10.1002/jts.20583

[CR34] Lahti, K., Vänskä, M., Qouta, S. R., Diab, S. Y., Perko, K., & Punamäki, R. L. (2019). Maternal experience of their infants’ crying in the context of war trauma: Determinants and consequences. *Infant Mental Health Journal*, *40*(2), 186–203. 10.1002/imhj.21768. 30715730 10.1002/imhj.21768

[CR35] Laor, N., Wolmer, L., & Cohen, D. J. (2001). Mothers’ functioning and children’s symptoms 5 years after a SCUD missile attack. *American Journal of Psychiatry*, *158*(7), 1020–1026. 10.1176/appi.ajp.158.7.1020. 11431222 10.1176/appi.ajp.158.7.1020

[CR36] Li, H., Bowen, A., Bowen, R., Balbuena, L., Feng, C., Bally, J., & Muhajarine, N. (2020). Mood instability during pregnancy and postpartum: A systematic review. *Archives of Women’s Mental Health*, *23*(1), 29–41. 10.1007/s00737-019-00956-6. 30834475 10.1007/s00737-019-00956-6

[CR37] Lieberman, A. F. (2011). Infants remember: War exposure, trauma, and attachment in young children and their mothers. *Journal of the American Academy of Child and Adolescent Psychiatry*, *50*(7), 640–641. 10.1016/j.jaac.2011.04.009. 21703490 10.1016/j.jaac.2011.04.009

[CR38] Mahler, M. S., & La Perriere, K. (1965). Mother-child interaction during separation-individuation. *The Psychoanalytic Quarterty, 34(4), 483–498*. 10.1080/21674086.1965.11926361. 5833244

[CR39] Martin, R. C., Bridgett, D. J., Mayes, L. C., & Rutherford, H. J. (2020). Maternal working memory, emotion regulation, and responsivity to infant distress. *Journal of Applied Developmental Psychology*, *71*, 101202. 10.1016/j.appdev.2020.101202.

[CR40] Masten, A. S. (2017). Building a translational science on children and youth affected by political violence and armed conflict: A commentary. *Development and Psychopathology*, *29*, 79–84. 10.1017/S0954579416001164. 27866496 10.1017/S0954579416001164

[CR41] Masten, A. S., & Narayan, A. J. (2012). Child development in the context of disaster, war, and terrorism: Pathways of risk and resilience. *Annual Review of Psychology*, *63*(1), 227–257. 10.1146/annurev-psych-120710-100356. 21943168 10.1146/annurev-psych-120710-100356 PMC5858878

[CR42] Mathiesen, K. S., Sanson, A., Stoolmiller, M., & Karevold, E. (2009). The nature and predictors of undercontrolled and internalizing problem trajectories across early childhood. *Journal of Abnormal Child Psychology*, *37*, 209–222. 10.1007/s10802-008-9268-y. 18766436 10.1007/s10802-008-9268-y

[CR43] Metzner, S., Verhey, J., Braak, P., & Hots, J. (2018). Auditory sensitivity in survivors of torture, political violence and flight—An exploratory study on risks and opportunities of music therapy. *Arts in Psychotherapy*, *58*, 33–41. 10.1016/j.aip.2018.02.001.

[CR44] Newman, D. A. (2014). Missing Data: Five practical guidelines. *Organizational Research Methods*, *17*(4), 372–411. 10.1177/1094428114548590.

[CR47] Pat-Horenczyk, R., & Schiff, M. (2019). Continuous traumatic stress and the life cycle: Exposure to repeated political violence in Israel. *Current Psychiatry Reports*, *21*(8). 10.1007/s11920-019-1060-x. 10.1007/s11920-019-1060-x 31264027

[CR45] Pat-Horenczyk, R., Achituv, M., Kagan Rubenstein, A., Khodabakhsh, A., Brom, D., & Chemtob, C. (2012). Growing up under fire: Building resilience in young children and parents exposed to ongoing missile attacks. *Journal of Child and Adolescent Trauma*, *5*(4), 303–314. 10.1080/19361521.2012.719595.

[CR48] Pat-Horenczyk, R., Ziv, Y., Asulin-Peretz, L., Achituv, M., Cohen, S., & Brom, D. (2013). Relational trauma in times of political violence: Continuous versus past traumatic stress. *Peace and Conflict: Journal of Peace Psychology*, *19*(2), 125–137. 10.1037/a0032488.

[CR46] Pat-Horenczyk, R., Cohen, S., Ziv, Y., Achituv, M., Asulin-peretz, L., Blanchard, T., Schiff, M., & Brom, D. (2015). Emotion regulation in mothers and young children faced with trauma. *Infant Mental Health Journal*, *36*(3), 337–348. 10.1002/imhj.21515. 25941026 10.1002/imhj.21515

[CR49] Punamäki, R. L. (2002). The uninvited guest of war enters childhood: Developmental and personality aspects of war and military violence. *Traumatology*, *8*(3), 181–204. 10.1023/A:1015211529584.

[CR50] Qouta, S., Punamäki, R. L., & Sarraj, E., E (2008). Child development and family mental health in war and military violence: The Palestinian experience. *International Journal of Behavioral Development*, *32*(4), 310–321. 10.1177/0165025408090973.

[CR51] Riem, M. M. E., Bakermans-Kranenburg, M. J., Pieper, S., Tops, M., Boksem, M. A. S., Vermeiren, R. R. J. M., Van Ijzendoorn, M. H., & Rombouts, S. A. R. B (2011). Oxytocin modulates amygdala, insula, and inferior frontal gyrus responses to infant crying: A randomized controlled trial. *Biological Psychiatry*, *70*(3), 291–297. 10.1016/j.biopsych.2011.02.006. 21470595 10.1016/j.biopsych.2011.02.006

[CR52] Rutherford, H. J., Booth, C. R., Luyten, P., Bridgett, D. J., & Mayes, L. C. (2015). Investigating the association between parental reflective functioning and distress tolerance in motherhood. *Infant Behavior and Development*, *40*, 54–63. 10.1016/j.infbeh.2015.04.005. 26025253 10.1016/j.infbeh.2015.04.005 PMC4526429

[CR53] Sadeh, A., Hen-Gal, S., & Tikotzky, L. (2008). Young children’s reactions to war-related stress: A survey and assessment of an innovative intervention. *Pediatrics*, *121*, 46–53. 10.1542/peds.2007-1348. 18166556 10.1542/peds.2007-1348

[CR54] Sadeh, A., Juda-Hanael, M., Livne-Karp, E., Kahn, M., Tikotzky, L., Anders, T. F., Calkins, S., & Sivan, Y. (2016). Low parental tolerance for infant crying: An underlying factor in infant sleep problems? *Journal of Sleep Research*, *25*(5), 501–507. 10.1111/jsr.12401. 26990152 10.1111/jsr.12401

[CR55] Sagi-Schwartz (2012). Children of War and Peace: A Human Development Perspective. *Journal of Conflict Resolution*, *56*(5), 933–951. 10.1177/0022002712446128.

[CR56] Sameroff, A. (2010). A unified theory of development: A dialectic integration of nature and nurture. *Child Development*, *81*(1), 6–22. 10.1111/j.1467-8624.2009.01378.x. 20331651 10.1111/j.1467-8624.2009.01378.x

[CR57] Slone, M., & Mann, S. (2016). Effects of war, terrorism and armed conflict on young children: A systematic review. *Child Psychiatry and Human Development*, *47*(6), 950–965. 10.1007/s10578-016-0626-7. 26781095 10.1007/s10578-016-0626-7

[CR58] Yahav, R. (2011). Exposure of children to war and terrorism: A review. *Journal of Child and Adolescent Trauma*, *4*(2), 90–108. 10.1080/19361521.2011.577395.

[CR59] Zamir, O., Gewirtz, A. H., Dekel, R., Lavi, T., & Tangir, G. (2020). Mothering under political violence: Post-traumatic symptoms, observed maternal parenting practices and child externalising behaviour. *International Journal of Psychology*, *55*(1), 123–132. 10.1002/ijop.12557. 30537100 10.1002/ijop.12557

[CR60] Zeifman, D. M. (2003). Predicting adult responses to infant distress: Adult characteristics associated with perceptions, emotional reactions, and timing of intervention. *Infant Mental Health Journal*, *24*(6), 597–612. 10.1002/imhj.10077.

[CR61] Zeskind, P. S., & Lester, B. M. (1978). Analysis of cry features in newborns with differential fetal growth. *Child Development*, *49*(3), 580–589. 10.2307/1128224. 7238145

